# Social Accountability in Maternal Health Services in the Far-Western Development Region in Nepal: An Exploratory Study

**DOI:** 10.15171/ijhpm.2019.05

**Published:** 2019-02-17

**Authors:** Mukesh Hamal, Kalina Heiter, Lian Schoenmakers, Myonne Smid, Tjard de Cock Buning, Vincent De Brouwere, Azucena Bardají, Chiranjibi Nepal, Marjolein Dieleman

**Affiliations:** ^1^Athena Institute for Research on Innovation and Communication in Health and Life Sciences (VU University), Amsterdam, The Netherlands.; ^2^Maternal and Reproductive Health, Department of Public Health, Institute of Tropical Medicine, Antwerp, Belgium.; ^3^ISGlobal, Hospital Clínic-Universitat de Barcelona, Barcelona, Spain.; ^4^CARE, Lalitpur, Nepal.; ^5^KIT Health, Amsterdam, The Netherlands

**Keywords:** Social Accountability, Governance, Maternal Health, Health Services, Nepal

## Abstract

**Background:** Social accountability or citizen-led accountability has been promoted in many low- and middle-income countries to improve the quality, access to and use of maternal health services. Experiences with social accountability in maternal health services in Nepal have not yet been documented. This study identifies existing social accountability structures and activities in maternal health services in two districts of Far-Western Nepal and explores their functions, implementation and gaps/challenges.

**Methods:** An exploratory study was conducted that included in-depth interviews with purposively selected policy advisors (8), healthcare officials (11), healthcare providers (12) and non-governmental staff (3); and focus group discussions (FGDs) with 54 women. Data analysis was conducted using thematic content analysis based on George’s *information, dialogue and negotiation* framework.

**Results:** Social accountability in maternal health existed in terms of structures such as mothers’ groups (MGs), female community health volunteers (FCHVs) and Health Facility Operation and Management Committees (HFOMCs); and *activities* such as social audits and community health score board (CHSB). MGs and FCHVs were perceived as trusted intermediaries, but their functioning was limited to *information*. HFOMCs were not fully functional. Social audits and CHSBs were implemented in limited sites and with poor participation by women. Health-sector responses were mainly found at the local level. Factors contributing to these challenges were the absence of a mandate and limited capacity, including resources.

**Conclusion:** Formal structures and activities existed for social accountability in maternal health services in the Far-Western Development Region of Nepal, but there were limitations pertaining to their implementation. The main recommendations are: for clear policy mandates on the social accountability roles of MGs and FCHVs; wider implementation of social audits and CHSBs, with emphasis on the participation of women from disadvantaged groups; improved capacity of HFOMCs; and improved engagement of the health sector at all levels to listen and respond to women’s concerns.

## Background

Maternal mortality is still a major concern in low- and middle-income countries such as Nepal. Despite remarkable progress in achieving the Millennium Development Goal of reducing maternal mortality, the country still has a high ratio (258 maternal deaths per 100 000 live births) compared to neighbouring countries, such as Bangladesh, Bhutan, and India.^1^ Access to and the quality of health services are crucial to achieve a significant reduction of maternal mortality.^2^ However, challenges related to access to and use of maternal health services persist, especially among ‘disadvantaged groups’ such as rural and socio-economically marginalized castes and ethnic groups in Nepal.^3,4^ For example, in addition to the lack of transportation, long distance to health facilities, and difficult geographical terrain in rural areas, health facilities often lack round-the-clock maternal health services, trained healthcare providers, equipment and drugs.^3,5-7^ Women have reported a lack of proper referral, unfriendly and discriminatory behaviours of healthcare providers, etc.^3^

Social accountability has been highlighted as one of the mechanisms that can improve the quality, access to and use of maternal health services.^8,9^ This refers to citizen-led accountability, in which citizens engage in exacting accountability from public sector actors such as politicians, policy-makers and service providers.^8,10,11^ Accountability basically refers to *answerability*, the obligation to inform and explain or justify one’s action, and *enforceability*, being subject to some form of sanction or reward based on the action.^12^ Social accountability mechanisms are thought to trigger responsiveness from the health sector to addresses the concerns and needs identified by citizens.^13^ This contributes to improving the quality, access to and use of services, and ultimately reducing maternal mortality.^8^

Efforts are being made by both the Government and civil society in Nepal to promote social accountability in the public health sector through the use of social accountability ‘mechanisms’ (structures, tools and activities/processes), such as health management committees, social audits and community health score boards (CHSBs).^3,14,15^ These initiatives aim to increase citizens’ capacity in the management and oversight of public health services. It remains unclear, however, whether these mechanisms function and how and if their aim is achieved. Except for a few studies that focused on specific activities, such as social audits, citizen charters and a suggestions/complaints box,^3,15,16^ we found no studies exploring social accountability mechanisms for maternal health services in Nepal, how they function, and their potential to address local concerns about health services. Social audits, implemented by the District (Public) Health Offices (D(P)HOs) and facilitated by non-governmental organizations (NGOs) at least once every year in a health facility, inform people about available health services and entitlements, and enhance mutual accountability and ownership of health services by communities and local health authorities.^3,15,16^ However, people are often not aware about their existence and therefore rarely participate in social audits, particularly in rural areas.^3^

The objective of this paper is to describe and discuss social accountability mechanisms that exist for maternal health services in 2 districts of the Far-Western Development Region of Nepal (hereafter Far-Western Nepal) using George’s^9^ *information*, *dialogue and negotiation* framework for accountability.

### Conceptual Framework

George identifies accountability as a mediator between actors in an unequal power relationship.^9^ In the health sector, this involves healthcare providers and health authorities as duty-bearers and citizens or patients/clients as rights-holders.^17^ Social accountability essentially entails contesting unequal power relationships through the empowerment of citizens, especially the disadvantaged groups.^9^ It includes representation of their voices in policies and programs and transformation of how they perceive themselves as well as how they are perceived by health-sector actors. These processes of empowerment, representation and transformation are achieved through *information*, *dialogue, and negotiation*.^9^

*Information* is a precondition for change as people cannot demand services and accountability if they do not know what they need and what they are entitled to.^9,10^ Different types of information in maternal health can be distinguished: (*i*) maternal healthcare needs and behaviour, (*ii*) rights and entitlements, and (*iii*) performance of the health sector, such as healthcare providers’ performance, implementation of health policies, etc. Citizen charters, for example, provide information about health entitlements, while audits provide information about the performance of health institutions. Acts guaranteeing a right to information, such as the 2007 Rights to Information Act in Nepal,^16^ enable the public to obtain information about health-sector performance. Information can generate awareness and support positive behaviours among women by reinforcing learning and dialogue.^9^

Once information is acquired, *dialogue and negotiation* form the next crucial step in generating change by mitigating social biases and overcoming barriers. Dialogues among health professionals can break down the existing misconceptions within the hierarchical health sector. Dialogues between health professionals and women can encourage them to reflect on prevailing negative assumptions and facilitate mutual understanding.^9,18^

Engaging marginalized groups in a participatory process of learning through information and dialogue can lead to a critical consciousness about the social drivers of their marginalization and facilitate the formulation of strategies to tackle them. This can also facilitate the formation of collective identities, creating a sense of group solidarity and of agency to confront an unequal power relationship.^10^

Efforts to facilitate dialogues between health workers and communities can lead to alliances between them.^9,10^ Such alliances can create an enabling environment for negotiation with the more powerful groups to acknowledge the needs and rights of the marginalized groups and allocate resources to them.^9,10^ Social accountability mechanisms should therefore facilitate the provision of information to citizens and dialogue and negotiation between citizens and health-sector actors to voice citizens’ concerns and hold the state and service providers accountable for their actions.

### Organization of Maternal Health Services in Nepal

Nepal is divided into 75 districts across three ecological zones (from north to south – mountain, hill and *terai*/plain region) and 5 development regions (Eastern, Central, Western, Mid-Western, and Far-Western).^19^ Under the country’s decentralized health system, districts form the basic unit for health planning and management.

Within a district, health posts are the first point of contact at the village level (Village Development Committee, VDC), followed by primary healthcare centres and health centres at the sub-district level, and district hospitals. Health posts extend community-based health activities through female community health volunteers (FCHVs) at the *ward* level (9 wards make 1 village) and outreach clinics.^20^

## Methods

### Study Setting

The study was conducted in 2 districts of Far-Western Nepal – Doti and Kailali. The districts were selected for their relatively poor maternal health status (eg, maternal mortality ratio of 263/100 000 in Kailali^4^) across the region, and feasibility of the study (availability of local support for data collection). The 2011 Demographic and Health Survey showed that this region had the lowest proportion of institutional deliveries (29.0%) compared to the national average of 35.3%.^21^ The low institutional delivery rates were explained by a lack of awareness among women and their family about its importance and poor access to health facilities.

Kailali is predominantly a *terai* district, most of whose population (72.2%) is urban^22^ while Doti is predominantly a hilly district with 67% of its population living in rural areas. Kailali has greater access than Doti to roads and highways and to transport. The indicators also show a better socio-economic status in Kailali than in Doti. For example, the female literacy rate is 57.1% for Kailali, while it is 42.3% for Doti.^23^

**Figure 1:**
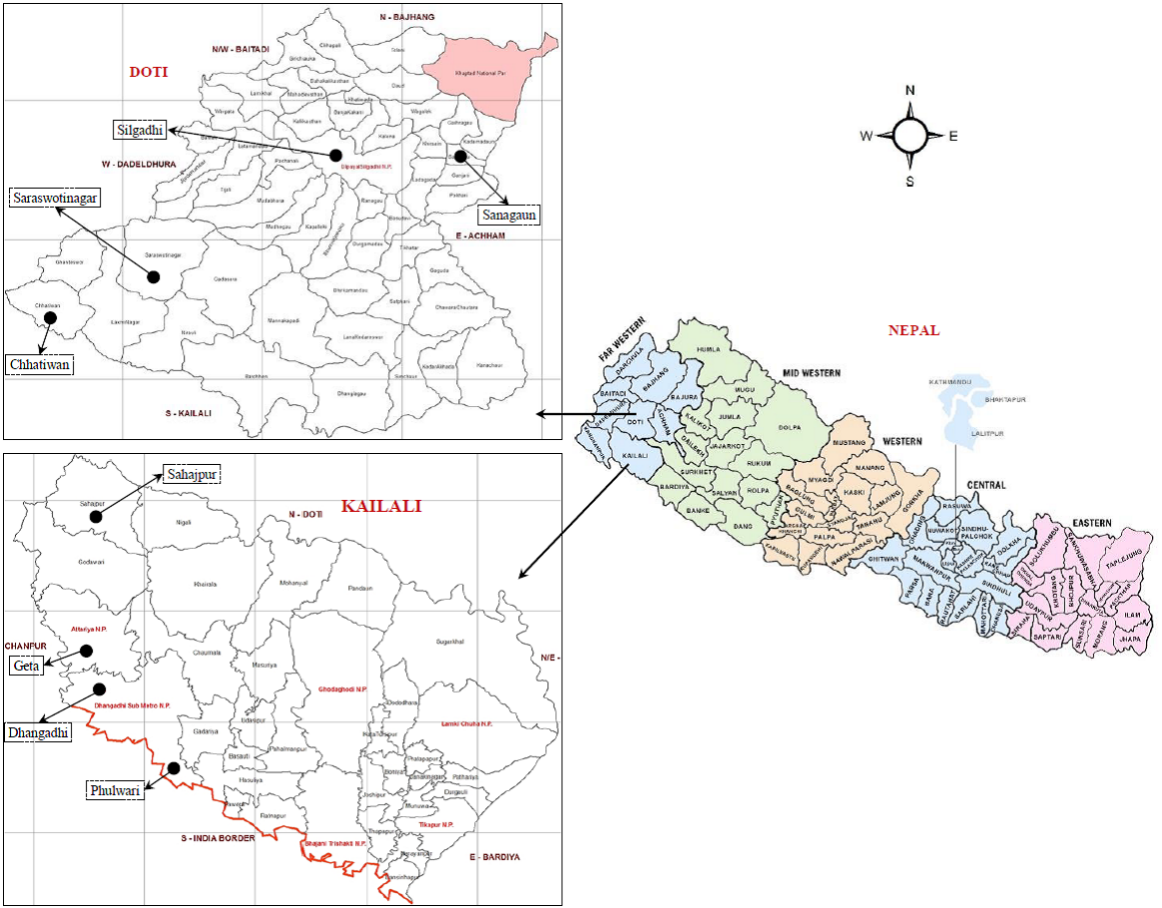
District Map of Far-Western region, Nepal With Study Sites. Source: Ministry of Federal Affairs and Local Development (MoFALD), Nepal^24^ and Local Nepal Today.^25^

The study was conducted in 4 sites from each district in consultation with CARE, an international NGO, to include both remote and less remote areas (Figure 1).

### Study Population, Sampling and Data Collection

The study population included all persons at the community, health facility and local government levels in the study sites and national-level policy advisors with an active role in public maternal health service delivery. Different respondent groups were identified, guided by the World Bank’s framework on accountability: *policy advisors, healthcare officials, healthcare providers* and* citizens*.^26^ We purposefully identified and recruited 88 respondents with rich information on maternal health services and social accountability^27^ and in consultation with CARE taking into account their availability at the time of data collection. At the VDC level, healthcare officials, healthcare providers and women were selected from the same service-provision areas to facilitate a complete understanding of a particular service delivery site and triangulation of the information collected. An overview of the study participants can be found in Table.

**Table:** Overview of Study Participants

**Respondent Category**	**District**		**Total**
	**Kailali**	**Doti**	
Policy advisors (national level)			
Government sector	4	4
Non-government sector	4	4
Healthcare officials			
District manager	3	2	5
Health Facility In-Charge^a^	3	2	5
HFOMC Member	0	1	1
Healthcare providers			
Doctor	0	2	2
ANM	1	4	5
FCHV	3	2	5
Citizens			
NGO staff	1	2	3
Women	33	21	54

Abbreviations: HFOMC, Health Facility Operation and Management Committee; ANM, auxiliary nurse-midwife; FCHV, female community health volunteer; NGO, Non-governmental organization. ^a^One Health Facility In-Charge each from Kailali and Doti is a doctor with a managerial role.

Data was collected through in-depth interviews with all respondents, plus focus group discussions (FGDs) with women. Interview and FGD guides were developed for each group of respondents, translated into Nepali and pre-tested. The in-depth interviews were aimed at identifying existing social mechanisms for maternal health services and their implementation and gaps/challenges, while the FGDs were focused on gaining an understanding of the women’s experiences and perspectives about social accountability mechanisms. The interviews and FGDs were audio-taped with prior consent from the participants.

Three researchers collected the data with trained local research assistants, who did the translations during the data collection. Two researchers collected data in April and May 2016. A third researcher collected additional data in April and May 2017, especially at the district level and below due to insufficient information collected in the first round. None of the research team members had any relationships with the study participants prior to the commencement of the research.

### Data Analysis

The recorded data was transcribed verbatim, and non-English transcripts were translated into English by the research assistants. Thematic content analysis was used and comprised a mix of inductive and deductive coding. Data analysis started with familiarization through reading and re-reading the transcripts individually. This was followed by coding the data in 2 steps: social accountability mechanisms existing at the study sites were identified, then themes were identified underlying each social accountability mechanisms based on the conceptual framework and the research questions. Emerging themes were given new codes.^27^ To ensure consistency of the coding among the researchers, each transcript was double-coded – after being done independently by each researcher, the coding was compared and discussed with another researcher to come to a consensus. MAXQDA 11 software was used for coding and organizing the data. Data collected from different categories of respondents on the same questions were triangulated, and differences were described and interpreted.^28^

## Results

This section starts with a description of the women’s voices and ways of communicating concerns to the health sector, followed by a presentation of the main social accountability structures and activities that exist at the study sites. Thereafter, challenges regarding the health sector’s responsiveness are presented.

### Women’s Voice Concerning Maternal Health Services

*Voice* refers to expressing needs and complaints regarding health services.^8^ Women complained during the FGDs about maternal health services, especially ones from the *terai* and less remote areas. While the women’s complaints were mainly about negative behaviours and mistreatment by healthcare providers, the healthcare officials and healthcare providers concerns were about the absence of healthcare providers at health facilities, difficulty in receiving timely care at health facilities, and not receiving government entitlements, such as medicines or maternity incentives, on time or at all.

“*First of all, they complain ‘your health workers don’t come on time in our health facility.’ Or they complain that they don’t get services early easily. Another complaint is that they don’t get extra medicines, they don’t get medicines. These three things*” [Healthcare Official, Kailali].

However, not all women voiced their concerns as mentioned in one FGD:

*“We just keep in our hearts.*”

Women in hilly and remote areas, on the other hand, sounded positive about the healthcare provided. Rather than complaints, they expressed expectations about additional services such as free medicines.

Some reasons why women did not complain about health services were: lack of awareness about their health rights, their shyness, and communities’ perception of healthcare providers as respected and important people.

“*Generally, in the rural areas community people are not empowered to complain directly to the health facility because they think those people working in health facilities are busy, very important persons, and they are not supposed to complain. That is the perception they have in rural areas because they are not aware of their rights”* [Policy Advisor, non-government].

### Communicating Concerns and Complaints to the Health Sector

Women in our study areas often mentioned communicating their maternal health concerns directly to the relevant healthcare providers. In most cases such communications were related to maternal health problems rather than complaints/concerns about maternal health services. Women mainly feared of reprisals if they complained directly to the healthcare providers.

“*People fear to complain because they think we might get angry when we hear about our complaints. They tell everything to FCHV and request them not to disclose their names*” [Healthcare provider, Kailali].

In contrast, a healthcare official (Kailali) explained that the existence of local healthcare providers and the community’s familiarity and closeness to them enabled people to make direct complaints to the healthcare providers. Another healthcare provider (Kailali) explained that the high staff turnover prevents women from building a relationship of trust with healthcare workers, which meant that they often do not feel safe when complaining to the healthcare provider.

**Figure 2:**
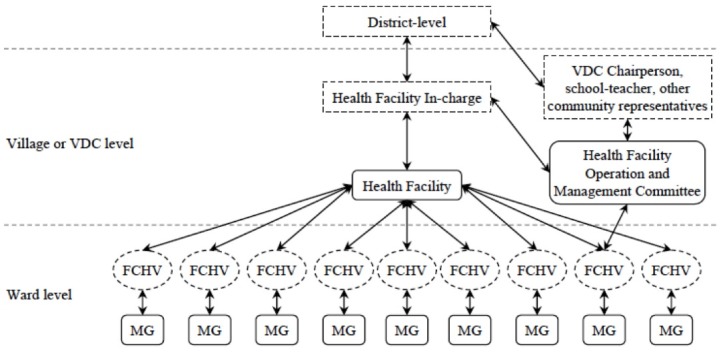
Organization of MG, FCHV, and HFOMC at Village/Ward-Level. Abbreviations: MG, Mothers’ group; FCHV, female community health volunteer; HFOMC, Health Facility Operations and Management Committee; VDC, Village Development Committee.

As an alternative, women used intermediaries such as mothers’ groups (MGs), FCHVs, Health Facility Operation and Management Committee (HFOMC) members to voice their concerns to the health sector, and they participated in activities such as social audits or CHSB. Box 1 provides an overview of these intermediaries, and Figure 2 visualizes the village/ward-level organization of MGs, FCHVs, and the HFOMCs.

**Box 1. Intermediaries Between Women and the Health Sector**

MGs and FCHV are formal, government-initiated basic structures for maternal health services at the community level and were established in 1988/1989.

*MGs* comprise all women of reproductive age in a *ward* interested in participating in the group, which undertakes local health and social activities. Priority is given to mothers with children under 5 years of age, pregnant and newly married women.^29,30^ Each MG selects a local *FCHV* to conduct *monthly meetings* and offer health-related information, which the MG members then share with other community members.^29^

*FCHVs* are self-motivated women of 25-45 years willing to serve the community, preferably literate and from the disadvantaged groups. They are trained to provide health education and promotion and basic curative services for minor health problems as well as referrals, primarily for maternal, neonatal and child health, family planning and selected infectious diseases.^29,30^ Some FCHVs also serve in the local health management committees known as HFOMCs.

*HFOMCs* were established in early 2000 as a part of the 1999 Local Self- Governance Act of the Nepal Government to promote community people’s engagement in health facility management and to hold health facilities accountable.^31,32^ Generally, there are 9-13 members in a committee, including the VDC or village chairperson, the Health Facility In-Charge, a school teacher, a FCHV, and community representatives.^33^ In addition to health facility management and oversight, HFOMCs are supposed to conduct a social audit annually and ensure awareness about the citizen charter.^34^ Citizen’s Charters are public-display boards that inform citizens about their rights and entitlements about public services, such as service availability, opening hours, service-related costs, procedures.^3^

Abbreviations: MGs, mothers’ groups; FCHVs, female community health volunteers; HFOMCs, Health Facility Operations and Management Committees; VDC, Village Development Committee.

Respondents also mentioned other social accountability mechanisms such as a suggestion box and public hearings. However, there was little information available about them in our study sites, potentially due to their limited implementation or use.

### Existing Social Accountability Structures and Activities

#### (i) Intermediary Structures

##### (a) Mothers’ Group and Female Community Health Volunteer

MGs and FCHVs were described in both districts as the most important structures for maternal healthcare as well as social accountability. MGs were reported to exist in all *wards* in the eight study sites, each group having 15 to 39 members.

Every MG had one FCHV selected by the members, except for some study sites (both urban and rural), where 14-40 FCHVs were reported per *ward* to serve a larger number of households or a large geographical area. MGs in almost all study sites met once a month. They conduct savings and credit activities and provided loans to the members who needed money for healthcare that they repay with interest. This was also the reason why some women joined the MG.

“*We have collected funds among ourselves to help those mothers who are in need in order to access all health facilities. So, we became the mothers’ group member*” [Women, Hill, Doti].

*Social accountability function* – FCHVs and MGs communicated the concerns of women to the health sector. Women discussed and shared their maternal health problems in the MG meetings, including their health service experiences and concerns. In these meetings, FCHVs provided *information* regarding maternal and child health and health services. FCHVs play a major role in linking communities with the health sector and in convincing them to use the health services. FCHVs were described as *mediators* between the communities and the health facilities, who also communicated *information* on women’s concerns about the health services to the health sector. Almost all healthcare providers and women mentioned FCHVs as their first point of contact to communicate their concerns and complaints about health-related matters, such as inappropriate behaviours of healthcare professionals.

Two healthcare officials (Kailali) explained that FCHVs, being female, were convenient persons for women to share their problems with.

“*It is easier for ladies to share their problems with ladies than to gents. And then FCHVs tell that* (problems) *to us.*”

FCHVs prepare a monthly report during the MG meeting, which includes the communities’ health service-related concerns. They submit and discuss this report in monthly meetings at the health facility attended by all health facility staff.

At some sites, FCHVs communicated women’s concerns directly to the healthcare officials and providers verbally. In some cases, they reported concerns directly to HFOMC, while in other cases they first reported concerns to the health facility, and when these were not resolved by the health facility, they went to the HFOMC. If these could not be resolved by the health facility or HFOMC, the concerns were taken to higher levels such as the D(P)HO.

One FCHV (Kailali) also explained:

“*If we have problem related to health, we go to health facility, but if we have problem related to administration or some management issue, then we go to HFOMC.*”

*Perceived changes* – Women from Kailali district felt that their complaints, mainly related to healthcare providers’ behaviour, were addressed when they were communicated.

“*Now with those coming to get the services, they are not talking rudely, that has improved”* [Women, Non-hill, Kailali].

Healthcare officials and providers from both *terai* and hills of Kailali district also mentioned that the complaints were acted upon.

*Gaps/challenges* – Even though MGs were described as a major platform where women shared and discussed their maternal health concerns, most of the examples of issues discussed in the MG meetings were related to the medical aspects of maternal health rather than health services. Moreover, it seemed that MGs were more often used for disseminating information about maternal health than for discussing the communities’ concerns about the health services. An NGO staff mentioned that not all women in the MGs, especially those with little education, can express themselves in the meetings.

The focus of the FCHV meetings seemed to be on monitoring and supervision of the FCHVs’ activities and less on addressing women’s health service concerns, as explained by one FCHV (Hill, Kailali):

“*Nothing like that (discussion on women’s complaints/concerns) happens, just submit the report, deposit fund (collected during the MG meeting) and then leave.”*

The MG meetings lacked opportunities for women to have *dialogues and negotiations* with the health-sector actors. Only at 4 sites were the MG meetings sometimes attended by health staff such as auxiliary nurse-midwives (ANMs) and nurses.

Other gaps/challenges that were mentioned included political influence on the selection of FCHVs, irregular MG meetings, issues of membership of MGs for some women, and expression of service needs in the meetings.

“*After political elections we get a lot of pressure to select FCHVs. (..) So, there is no equal chance for all FCHVs to be selected. But this is for some FCHVs only. Sometimes we wonder how the FCHVs were selected, since they don’t have education or knowledge and are not good at their job*” [Healthcare Official, Doti].

Some women (not MG members) said they did not attend the meetings due to distance or being busy with household chores.

##### (b) Health Facility Operation and Management Committee

HFOMCs were reported to exist in all health facilities of the study sites each having 8-11 members. Most HFOMCs conduct monthly meetings or more often in case of an emergency.

*Social accountability function* – HFOMCs play a role in *information* provision as well as *dialogue and negotiation*. HFOMCs receive information on women’s needs and concerns from the committee members, mainly the FCHVs and health facility staff. The policy advisors (non-government) particularly highlighted that locally elected representatives of women and marginalized ethnic groups, who are HFOMC members, voiced the concerns of these groups in the committee meetings. These concerns pertained to healthcare providers’ behaviour and lack of equipment and supplies, for instance.

“*In some health facilities, some people are demanding lab facility, additional ANM, nurses, staff. So, in that meeting Health Facility In-Charge discusses all those problems whether it is related to equipment, health problems, facilities etc”* [NGO Staff].

The HFOMC members encourage *dialogue and negotiation* among committee members and with other health facility staff in addressing the communities’ concerns. According to the respondents, all committee members participated equally in making decisions in the meetings.

“*Every member in HFOMC has equal rights. There is not any one leader who takes decisions. If any problem has occurred, the entire committee discusses and concludes”* [Healthcare Official, Doti].

The policy advisors (non-government) also explained the role of HFOMCs in holding the healthcare providers accountable and that they are mandated to do this by demanding explanations from them.

“*They* (HFOMCs) *are essential to manage and operate health facility and also to make health workers accountable to the community. Because we get lot of complaints of health workers’ absenteeism. And if health workers are not staying at the health facility, the management committee has the right to ask them, ‘Why are you leaving the post without notifying us? If you are going on a training make sure that the information is passed on, make sure alternate health worker is available to deliver services.’ So, this creates a mechanism to make those health workers accountable at the local level”* [Policy Advisor, non-government].

Respondents reported that HFOMCs relayed the information to the respective health facilities and/or district health authorities when they could not resolve the concerns. They also communicated the health service concerns to other authorities at the local level beyond health sector, such as the education sector or the development sector to address them.

*Perceived changes* – HFOMCs mostly utilized their own resources, eg, their health budget, or identified and mobilized other local resources in addressing communities’ needs and concerns.

“*For example, there was no birthing centre here before and there was no lab. Now on the initiative of the HFOMC and political parties, a lab is here. And for the birthing centre, this building was built by the VDC and DDC* (village- and district-level councils, respectively) *(..) Then for the birthing centre the VDC also recruited one ANM on their own salary*” [Healthcare Provider, Kailali].

Some healthcare officials described instances in which healthcare providers benefitted while addressing communities’ concerns, eg, recruiting an office assistant for security purposes, especially for female staff during night shifts.

According to the respondents, HFOMCs were ‘the backbone of health facilities’, playing a vital role in improving the health facilities and services.

*Gaps/challenges* – A gap some respondents mentioned was the lack of *active* and *well-represented* HFOMCs for better maternal health. A non-government policy advisor shared his general experience that the members were not always aware of their membership and were therefore inactive. An NGO staff (Kailali) mentioned a challenge concerning representation of 4 female members in HFOMCs as mandated by the national guideline. Our study however does not have any information on composition of HFOMCs in the study sites. A respondent from Doti described the high degree of influence of political patronage over the decisions concerning the health facility management and operations. Lastly, there was very little awareness of HFOMCs among women, especially in hilly areas, eg, women and even some ANMs in Doti were not aware about HFOMCs or their work.

### (ii) Activities

#### (a) Social Audit

Social audits were the government’s activities and were conducted/facilitated by independent NGOs, recruited through an open-tender process, in selected health facilities of both districts. For instance, in Kailali, they were reported being conducted in 28 (out of 41^35^) health facilities. A policy advisor (non-government) explained:

“*The District Health Office decides where it makes most sense to do that* (social audit)*. It’s based on the need.”*

Where conducted, they occur over a 5-day long process covering all health programs and services of a health facility usually for the last fiscal year.

*Social accountability function* – The overall process of a social audit involved both *information* and *dialogue and negotiation.* The *information* aspect included: collecting information on service provision through health facility data and interviews and FGDs with the health facility staff and communities, and sharing of the collected information in a public meeting.

“*The local NGO, they conduct survey, focus group discussion (..) the reality – how is the service from the facility. They do the auditing from demand-side perspective, community perspective”* [Policy Advisor, non-government].

The participants in the public meeting included: the health facility staff, FCHV and HFOMC members, beneficiaries, local community leaders and other key stakeholders such as teachers and the media. The meetings were also attended by the district-level authorities and sometimes the higher-level authorities such as the Regional Health Directorate.

“*MGs, FCHV, and HFOMC present their work for the whole year, their experience about the services they provided, and the problems and improvements (in the services). The press is also involved while presenting these results”* [Healthcare Provider, Doti].

The *dialogue and negotiation* aspect included the interactions in the public meeting: beneficiaries asked questions about health services, made demands and even complained, such as about the health staff behaviour, and service providers were required to answer their questions.

During the interactions, there were opportunities for both service providers and communities to clarify their actions.

“*If somebody did not receive, eg, maternity incentive, they have to question. But why is that? And they* (health service provides) *will also present the challenges from their side, and there will be time for beneficiaries to comment and raise questions. (..) So, it’s a two-way communication between the beneficiaries and the providers*” [Policy Advisor, non-government].

At the end of the social audit process, action plans are developed to address obstacles and constraints of the health service delivery agreed upon by both the community and the healthcare providers. These concerns and action plans were also forwarded to the district and higher levels, and reviewed and referred for necessary actions. A policy advisor (non-government) explained that the process of social audit follows the ‘win-win’ and ‘do no harm’ principles through a ‘not to blame each other’ approach, with both parties jointly addressing the problems of health service delivery. At one site (Doti), the involvement of the press (media) in publishing the results was seen as a vital aspect of the social audits by increasing the pressure for future improvements.

*Perceived changes* – Respondents explained that the social audits increased the communities’ awareness and interest in local health services. The communities’ concerns and interests helped healthcare providers to understand the needs of the community, thereby improving the quality of the services.

“*It has been helping a lot. All complaints they express during social audit (..) it helps us to stay aware. The behaviours we do unconsciously when we hear from public, we also get chance to improve that* (behaviour)*. (..) In some cases I also apologised because of the complaints (..)”* [Healthcare Provider, Kailali].

Two healthcare providers (Kailali) appreciated the effectiveness of the social audit in terms of learning and improving and ensuring accountability of the health sector actors. Women in 2 sites (Doti) felt that when they shared their problems during social audits, some of them were addressed. These changes only concerned local-level actions. Other healthcare providers, women or respondents never mentioned about effectiveness of social audits.

*Gaps/challenges* – Firstly, the social audits were not conducted in all health facilities. A healthcare official mentioned that this was mainly due to budget constraints. A policy advisor (non-government) explained:

“*Actually, last year there was a proposal from the Ministry of Health to the Ministry of Finance to include these activities* (social audits)*, but the Ministry of Finance cut these activities because of budget constraints.”*

Secondly, local women in some study sites did not know about the audits. Thirdly, a woman (Doti) and a policy advisor (non-government) mentioned that participation was limited in the audits, especially by women from marginalized castes, either in terms of attendance or in terms of expressing themselves.

“*Women here cannot speak up, they are shy, (..) women here cannot speak up in front of all”* [Women, Doti].

Fourthly, according to a policy advisor (non-government), there was challenge in implementing the action plans of social audits due to a lack of technical and financial support, especially from the district and Ministry.

Lastly, we did not find any evidence of mechanisms to monitor and follow up the action plans once they were forwarded to higher levels. This particularly had implications on responses from higher levels.

#### (b) Community Health Score Board

Since 2011, CHSBs have been implemented by CARE in selected health facilities of the study districts in collaboration with the D(P)HO, local health facilities and HFOMCs. The health facilities were selected in consultation with the D(P)HO based on the poor status of maternal health. Unlike social audits that focused on the overall health services of a health facility, the CHSB focused on maternal and neonatal health.

*Social accountability functions* – The CHSBs also involved *information* and *dialogue and negotiation*. The *information* aspect concerned soliciting information on users’ perception of access, utilization and quality of maternal and neonatal health services of a health facility through the health facility data and FGDs with FCHVs, HFOMC and the communities, including MGs from remote and hard-to-reach areas. This information was then shared in a public mass meeting and was also used to select 12 indicators to be rated later in the public mass meeting. The meetings were attended by the health facility staff, HFOMC members, FCHVs, women and local stakeholders such as leaders and politicians.

The public mass meeting functioned as an *interface meeting* where the indicators were rated through *dialogue and negotiation* between the health service providers and community members. This involved negotiating scores together with justification and evidence from the health service providers and the community members.

“*HFOMC’s meeting, if this is the indicator we are talking about, we check everything like their meeting minutes. The Health Post In-Charge, he has to say, but he cannot only say, he has to provide proof if the meetings were held or not”* [NGO Staff].

Then, through a similar process of *dialogue and negotiation*, targets were set for the indicators for a given period, which included selecting and negotiating priorities in terms of activities to address gaps and reach targets, and allocating responsibilities among the health providers, community members and other stakeholders such as VDC or local leaders. The scores, targets and activities were reviewed and followed up every 6 months in a public mass meeting.

“*We have our reviews, how was it then and how is it now. For that, there is marking. Communities themselves do the marking, we do not do (..). In that way it has been easier to identify their needs and if they are satisfied with the services we have provided. From that we can know what we need to improve”* [Healthcare Official, Kailali].

The NGO facilitators were reported to ensure that everybody engaged in the entire process of the dialogue and negotiation.

“*We enable them to speak. We have to facilitate, otherwise they do not speak. Those who are from the marginalized, socially secluded groups, they hesitate to speak in such meetings”* [NGO staff].

The positive influence of groups over individual powerful actors (eg, political leaders) to enable the less-powerful actors (eg, the disadvantaged groups) in these events was particularly mentioned:

“*Political representatives are the minority, the majority are from MGs, FCHV”* [NGO Staff].

*Perceived changes* – CHSBs were reported to have led to positive changes in the health facilities and health services utilization by women. These changes were reported in terms of: decreased absenteeism among the health facility staff; increased concern among healthcare providers for mothers’ health and their children’s health; improved acceptance of management responsibilities of health facilities by the HFOMC members; generation of resources from local stakeholders such as VDC, NGOs to improve services at health facilities, eg, birthing centre; and increased awareness among women about maternal and neonatal health.

NGO staff explained that the health worker’s increased concern about women and their children’s health and the feeling that the health facilities are there for them improved women’s acceptance of and trust in the health services. They claimed that this trust also contributed to an increase in the use of maternal health services, such as institutional deliveries and antenatal and postnatal check-ups, as confirmed by the score boards.

*Gaps/challenges* – The first challenge of the CHSBs is that their implementation is limited to selected VDCs. Secondly, not all women attended the CHSBs, especially from hilly and remote areas. Their reasons included long distance to the meeting venue, lack of time, lack of information about the event and limited interest. Women in one site (Doti) mentioned that often the same women were invited to the CHSB.

Thirdly, the fact that the HFOMC Chair is a government employee is a challenge, as 2 NGO staff explained:

“*He can be transferred from one VDC to another. Once he is transferred, everything is gone.”*

Fourthly, continuation of the CHSB after the NGO’s program phase-out. A district-level healthcare official, referring to programs of the NGOs in general, stated:

“*They come for a time being and do not continue, they phase out after 2 years, three years. They discontinue. (..) We get support only when they are there, after they discontinue we are then back again in our regular situation.*”

Gaps/Challenges With Health Sector’s Responsiveness

A major gap concerned the lack of responses, especially from the higher levels such as the district and Ministry. Healthcare officials and healthcare providers mentioned sharing communities’ health service concerns in review meetings held regularly at the district level as well as other platforms such as higher-level visits, trainings and workshops. But they were worried that these authorities do not take the concerns seriously, and that the district health authorities refer them to other actors.

“*The District Health Office asks us to tell local party representative or management persons, who don’t take it seriously. No matter how many times we tell them, they never care. It is already three months and they haven’t responded. We do our work as it is”* [Healthcare Provider, Doti].

When asked why women do not complain about their health service concerns to the district authorities such as D(P)HO, a woman (Kailali, *Terai*) said:

"* Such high positioned people only talk, they don’t do what they say.”*

Three policy advisors – from the government and the civil society – and a district-level healthcare official particularly highlighted that the major reason for the lack of a proper response to communities’ concerns was a lack of resources, most commonly financial resources. They mentioned the overall budget allocated for health at the national level was very low. This was the reason mentioned by the district-level healthcare official about referring the communities’ health service concerns back to the HFOMC.

District-level and almost all other healthcare officials and healthcare providers commented on the late disbursement of the budget from the centre, which constrained them from conducting health programs as well as distributing maternal health benefits such as maternity incentives.

“*Our fiscal year starts from Shrawan* (mid-July in Nepali calendar)*, but we get our budget only in Mangsir/Poush* (mid-November/mid-December)*. But we have to continue providing services from Shrawan. That’s why we have problems”* [Healthcare Official, Kailali].

A policy advisor (non-government) stressed the district health authorities’ lack of capacity to listen to and address the communities’ health services concerns. Referring to the monthly meetings of the D(P)HO, the policy advisor mentioned that such concerns and complaints were presented, but they were neither analysed nor properly discussed. The policy advisor (non-government) explained that the paper-based monitoring system of the country constrained the provision of timely and accurate information needed to prioritize areas and resources for intervention, both at the district and national levels.

Another challenge at the district level was related to poor monitoring of the social accountability activities mainly due to limited personnel.

A healthcare official (Kailali) stated that there was a gap in monitoring and supervision, mainly from the national level. A policy advisor (non-government) also stated that the supervision and monitoring from the higher level were not continuous or functional.

## Discussion and Conclusion

Overall, our study showed that formal government structures for social accountability in maternal health services, namely MGs, FCHVs, and HFOMCs, exist in all study sites and have an intermediary linkage role. The government conducts social audits, and the NGOs conducted CHSBs through the health facilities and the HFOMCs in selected sites in both districts.

We found that although the intermediary structures are supposed to assume social accountability functions through information, dialogue and negotiation, in practice, their function seems to be limited to information provision, particularly raising awareness among women about maternal health services and entitlements and to some extent sharing women’s concerns with healthcare providers. This is due to a lack of a mandate and of political capabilities among women (explained below in *Gaps/Challenges* section), which means that these structures have the potential to play their linkage role better when they receive support and an official mandate and when women are helped to improve their political capabilities. The social accountability activities led to responses from the health sector. However, implementation of these activities was limited to a few health facilities, and only a few women participated. Health sector responses were mainly local, with limited responsiveness from the higher levels. This means that they have a potential for success, but this needs to be reinforced. This finding is ubiquitous in the social accountability literature.

The next sections reflect on these social accountability structures and activities and discuss mechanisms of influence, gaps and challenges as well as policy implications.

*Mechanisms of influence* – The social accountability structures and activities in the study sites enhanced the *answerability* as well as *enforceability* (to a limited extent) functions of accountability. In addition to creating awareness among women about maternal healthcare, services and state entitlements, they generated information and evidence about the health sector performance in terms of feedback on perceived quality and gaps in maternal health services from community members, and in particular from women. The feedback was communicated to the health sector mainly through public health meetings. It generated answerability as poor performance was often discussed and justified by the involved healthcare providers during the social audits and CHSBs.

Both structures and activities generated *enforceability* through informal sanctions, such as fear of repercussions among health-sector actors, improved monitoring and follow-up of women’s issues communicated to the health sector by healthcare officials and HFOMC members, albeit to a limited extent. This is in line with other studies, for instance, Lodenstein et al and Brinkerhoff explained that negative publicity or public exposure of poor performance in public forums creates incentives among public sector actors to respond positively because of a fear of repercussions.^13,36^ Involvement of the media in such events is likely to increase publicity about the problem, amplifying demands and spurring attention and follow-up for the public sector response.^10,17,37^

The social audits and CHSBs also enabled women to have a group dialogue and negotiation directly with the health sector. Schaaf et al and George showed that a group/collective approach protects individuals from risks that they might face when confronting powerful actors on their own.^9,38^ The role of an external facilitator was particularly crucial in mediating power relations between the women (less powerful) and the health sector (the more powerful).

The *dialogue and negotiation* between the women and the health-sector actors in our study facilitated collaborative solutions that benefitted both.^9^ Findings from our study corroborate evidence on social accountability activities which stimulated interactions between communities and healthcare providers in Nepal^3,15,16^ and other countries such as India,^10^ Guinea,^39^ Uganda,^40^ and Malawi.^18,41^ These activities provided expanded and inclusive spaces for the duty-bearers and the rights-holders to come together, share concerns (*information*), understand gaps and challenges in the health services, think through solutions and negotiate joint action plans (*dialogue and negotiation*).^16,18,40,41^ These activities helped to resolve problems with the health services locally, often with little or no external support. They also helped to build trust and strengthen the relationship between the community and service providers and generate responsiveness from the service providers.^16,18,40,41^

The social accountability structures had limited effectiveness: although trusted intermediaries such as FCHVs and HFOMC members reported concerns during regular meetings with healthcare providers and enabled women to have a dialogue and negotiation with the health sector through their representatives, thus indirectly, their functioning was hampered for several reasons as explained below.

*Gaps/challenges* – A major gap concerned a lack of a role of MGs and FCHVs in *dialogue and negotiation* with the health sector possibly in part due to a lack of a clear policy mandate for this role. For instance, unlike FCHVs, Accredited Social Health Activists, a similar cadre of community health volunteers in India, have a clear policy mandate for their role as an ‘activist’, ie, to create awareness among communities and mobilize them for local health planning and accountability, in addition to 2 other roles like that of the FCHVs, ie, ‘link worker’ and ‘service extension worker.’^42^ Lodenstein et al particularly highlighted the importance of a legal mandate for the accountability role as they found that the lack of this role was one of the major factors limiting the health facility committees in Guinea and Democratic Republic of the Congo from being socially accountable.^39^ Scott and Shanker, however, stressed that policy mandate alone is not sufficient to achieve a policy objective, and other structural obstacles, such as institutional support, remuneration, also need to be addressed.^43^

An important gap that hampers the effectiveness of any social accountability activity or structure is the lack of support to improve the *political capabilities* of the women in our study sites. For instance, an NGO in Uttar Pradesh in India enhanced political capabilities by engaging socially marginalized women in political action to advocate for their maternal health rights and demand accountability from the health sector through their grassroots organization “*Women’s Health Right Forum.*”^17^ According to George, political capabilities and political actions are particularly necessary to confront unequal power relationships and address drivers of social deprivation.^9^ We did not identify any support given to women in our study sites for actions on accountability and advocacy.

A third gap is related to the lack of responsiveness, especially from the higher levels in the health sector. Other studies^3,15,16,18,40,41^ also observed that the responses generated by social accountability structures and activities were limited to the local level. Gullo et al^18^ explained that the reason for this failure was a lack of sufficient emphasis on higher-level engagement during the accountability process. Even though we interviewed authorities at the national level, the focus of the social accountability structures and activities in our study was limited to the local level.

*Policy implications* – For the formal social accountability structures to function in a sustainable way and use their intermediary position optimally, they need to be reinforced, both legally and in their capacity to perform social accountability function: MGs and FCHVs were the most trusted structures for women to share and discuss their health service concerns. Providing these structures with a clear legal mandate to ensure heath sector accountability could aid in institutionalizing the process of mobilizing the MGs for social accountability. A focus should be placed on increasing their political capabilities for social accountability rather than their mere participation in social accountability activities organized by the government or NGOs. Additionally, an equal emphasis should be given to the supply-side, eg, training, delegation of authority, in such way that coherent policy will nurture actions at local level by legitimating changes at implementation level.^37,44^

HFOMCs also have a crucial role in the overall social accountability process, including organizing activities such as social audits and CHSBs and responding to communities’ concerns. Therefore, their members need to be active, and they should be aware of and have the capacity to perform their accountability roles. Disadvantaged groups should be well represented on these committees.

To address the lack of health sector responsiveness particularly at the higher levels requires that social accountability mechanisms function at multiple levels.^17,39^ Hamal et al suggested that accountability problems in health systems are systemic and addressing them requires a *systemic* approach.^45^ This could be done by: engaging higher-level state actors through strengthened state–society interaction; systematically shifting the incentives of state actors to respond to social accountability; and enforcing legal mechanisms to ensure the state actors abide by their ‘social contract.’^46^

## Acknowledgements

This manuscript is a part of the first author’s Erasmus Mundus Joint Doctorate (EMJD) program under International Doctorate in Transdisciplinary Global Health Solutions funded by European Commission (Erasmus Mundus Joint Doctorate Specific Grant Agreement 2013-1479). The authors would like to acknowledge the European Commission for providing the scholarship for the doctoral program. The authors also extend gratitude to the research assistants, all staff from CARE and its partner NGOs, and all other who supported in collection of the data. We would also like to thank the participants for their time and sharing their experiences and perspectives.

## Ethical issues

The technical approval for the study was granted by the Science Committee of the VU University Medical Center (VUmc) EMGO+ Institute in the Netherlands (WC2014-035 HZ). The ethical approval for data collection in Nepal was obtained from the Nepal Health Research Council (NHRC) (Reg. no. 22/2016). Participants were asked to confirm their consent and were assured of their privacy and confidentiality prior to the interviews or FGDs.

## Competing interests

Authors declare that they have no competing interests.

## Authors’ contributions

MH designed the overall study, coordinated the data collections, guided the analyses, and prepared the later draft of the manuscript. LS and MS designed the study, collected and analysed the data for Kailali and Doti, respectively. KH designed the subsequent study for both districts and collected and analysed the data. LS and KH also prepared the initial drafts of the manuscript for the data they collected. MD and TdCB advised on the initial design of the manuscripts and provided feedback on subsequent revisions. CN provided field level supervision for data collection, and reviewed and provided feedback on the later versions of the manuscript. VDB and AB reviewed and provided feedback on the later versions of the manuscript. All authors read and approved the final manuscript.

## Authors’ affiliations

^1^Athena Institute for Research on Innovation and Communication in Health and Life Sciences (VU University), Amsterdam, The Netherlands. ^2^Maternal and Reproductive Health, Department of Public Health, Institute of Tropical Medicine, Antwerp, Belgium. ^3^ISGlobal, Hospital Clínic-Universitat de Barcelona, Barcelona, Spain. ^4^CARE, Lalitpur, Nepal. ^5^KIT Health, Amsterdam, The Netherlands

## Key messages

**Implications for policy makers**

- Mothers’ groups (MGs) and female community health volunteers (FCHVs) are trusted representatives of women and require a clear mandate; their political capabilities need to be improved in order to play an active role in social accountability.
- Although Health Facility Operation and Management Committees (HFOMCs) have clear mandates and access to higher-level decision-makers, they require better awareness and capacity to perform their accountability roles.
- Social audits and community health score boards (CHSBs) are effective social accountability activities that need to be implemented in all areas, with wider participation of women, especially from the disadvantaged groups.
- The health sector, including the district level and above, requires engagement to listen and respond to women’s concerns.

**Implications for public**

Improving access to and use of maternal health services requires that the health sector be responsive to women’s concerns. Our study showed that women share their health service concerns in mothers’ groups (MGs) and with female community health volunteers (FCHVs), who have the potential to serve as intermediaries between the women and the health sector. To use this potential, these structures need a clear mandate and improved political capabilities. The Health Facility Operation and Management Committees (HFOMCs) have an important function in social accountability. But to use their full potential, these committees require better interaction with the health sector, which needs better engagement at all levels to improve their responsiveness to women’s concerns.
